# A Genome-Wide Association Study on Feed Efficiency Related Traits in Landrace Pigs

**DOI:** 10.3389/fgene.2020.00692

**Published:** 2020-07-03

**Authors:** Lu Fu, Yao Jiang, Chonglong Wang, Mengran Mei, Ziwen Zhou, Yifan Jiang, Hailiang Song, Xiangdong Ding

**Affiliations:** ^1^National Engineering Laboratory for Animal Breeding, Laboratory of Animal Genetics, Breeding and Reproduction, Ministry of Agriculture, College of Animal Science and Technology, China Agricultural University, Beijing, China; ^2^Institute of Animal Husbandry and Veterinary Medicine, Anhui Academy of Agricultural Sciences, Hefei, China

**Keywords:** genome-wide association study, feed efficiency, feed conversion ratio, average daily gain, average daily feed intake, residual feed intake

## Abstract

Feed efficiency (FE) traits in pigs are of utmost economic importance. Genetic improvement of FE related traits in pigs might significantly reduce production cost and energy consumption. Hence, our study aimed at identifying SNPs and candidate genes associated with FE related traits, including feed conversion ratio (FCR), average daily gain (ADG), average daily feed intake (ADFI), and residual feed intake (RFI). A genome-wide association study (GWAS) was performed for the four FE related traits in 296 Landrace pigs genotyped with PorcineSNP50 BeadChip. Two different single-trait methods, single SNP linear model GWAS (LM-GWAS) and single-step GWAS (ssGWAS), were implemented. Our results showed that the two methods showed high consistency with respect to SNP identification. A total of 32 common significant SNPs associated with the four FE related traits were identified. Bioinformatics analysis revealed eight common QTL regions, of which three QTL regions related to ADFI and RFI traits were overlapped. Gene ontology analysis revealed six common candidate genes (*PRELID2, GPER1, PDX1, TEX2, PLCL2, ICAM2*) relevant for the four FE related traits. These genes are involved in the processes of fat synthesis and decomposition, lipid transport process, insulin metabolism, among others. Our results provide, new insights into the genetic mechanisms and candidate function genes of FE related traits in pigs. However, further investigations to validate these results are warranted.

## Introduction

Feed accounts for about 65% of the total cost in modern pig production and feed efficiency (FE) traits in pigs are critical (Sanchez et al., 2017). Better FE dramatically reduces production costs, thus contributes to a reduction of the final cost of products and decreases farming expenses and energy consumption (Ding et al., 2018). Breeding programs to improve FE have been undertaken for many years, but FE related traits, such as average daily feed intake (ADFI) and residual feed intake (RFI), are still difficult to be improved because they can neither be selected nor directly measured (Silva et al., 2019). Usually, FE is evaluated by four traits: feed conversion ratio (FCR), average daily gain (ADG), ADFI, and RFI (Onteru et al., 2013). The phenotypic measurements of FCR, ADFI, and RFI are difficult and costly which need an automatic feeding system. Besides, the selection of single FE related traits may affect other traits that are valuable for pig production, such as growth rate (Horodyska et al., 2017). This conundrum makes the genetic investigation of FE very important.

Therefore, it is essential to understand the molecular mechanism and genetic basis underlying FE related traits for the improvement of FE. In past decades, hundreds of quantitative trait loci (QTLs) affecting complex traits in pigs, including FE related traits, have been detected. Among these, 346, 618, 96, and 96 QTLs for FCR, ADG, ADFI, and RFI have been identified in different pig populations,^1^ respectively. However, their molecular regulation mechanism remains largely unknown.

In recent years, with the development of dense genomic markers and significant reduction in cost, genome-wide association study (GWAS) has become increasingly popular for detecting genetic variants associated with economic traits (Do et al., 2014b; Ding et al., 2018). Studies have revealed many potential candidate genes for FE related traits in pigs. For FCR, several researchers reported that significant SNPs and QTLs were mainly located on chromosomes (SSC) 4, 6, 7, 8, 17, and 18 (Sahana et al., 2013; Horodyska et al., 2017). Another study identified three QTLs for ADG, which were located on SSC 4, 14, and 15 (Ji et al., 2019). Compared to FCR and ADG, only few studies on RFI were carried out in pigs. Onteru et al. (2013) reported several QTLs on chromosomes 7 and 14 that are related to RFI in Yorkshire pigs. Recently, Silva et al. (2019) identified three QTL regions located on SSC1 that are associated with ADFI. Although high density chips and GWAS had detected more and more genetic variants in pig economic traits, FE related traits (ADFI and RFI) still progress slowly because of the difficultly in phenotype measuring and recording. In addition, most of these studies, mainly focus on Duroc and Yorkshire breeds, and FE related traits studies on Landrace have been rarely reported. So far, according to PigQTLdb,^1^ four FE related traits (FCR, ADG, ADFI, and RFI) have been reported 371 QTLs in Duroc, 185 in Yorkshire and only 46 in Landrace pigs. Therefore, further investigation is needed to better understand FE related traits in Landrace population.

In this study, we performed a GWAS in a Landrace population to identify genomic regions and genes associated with four FE related traits: FCR, ADG, ADFI, and RFI.

## Materials and Methods

### Ethics Statement

The whole recording procedure of ear tissue samples was carried out in strict accordance with the protocol approved by the Institutional Animal Care and Use Committee (IACUC) at the China Agricultural University. The IACUC of the China Agricultural University approved this study (permit number DK996).

### Animals and Phenotypes

In this study 296 Landrace pigs were sampled. Phenotypic information of two batches comprising 156 and 140 pigs was recorded from April to July, and July to August in 2018, respectively. The first batch of 156 pigs was obtained from 46 litters born in April (one to nine individuals from each litter with an average of three), and the 140 pigs in the second batch born in July were obtained from 41 L (one to eight individuals from each litter with an average of three). The original feeding records were automatically generated by the pig automatic feeding system (ACEMO128 Feeding station, France). The phenotypic data comprised individual ID, starting weight, daily feed intake, daily weight gain, final weight, feeding period, and feed conversion rate. Data were collected from each pig during the feeding period (approximately 11 weeks old), from 25 to 100 kg body weight (BW). The piglets were group-housed in half-open cement-floor pens (10–12 animals in each pen, with an average space of two m^2^ per pig). Each animal was labeled with a unique electronic identification tag on the ear and detected by the automatic feeding system. Once a pig visited the feeder, the date and exact start and stop feeding time, the animal number, and feed consumption of each visit were recorded.

The traits (ADFI, FCR, and ADG) for each pig were calculated throughout the testing periods according to the information provided by the automatic feeding system. ADFI was calculated by the total amount of recorded feed intake divided by the length of the fattening period. ADG was calculated by total weight gain (final weight minus initial weight) during measure periods divided by the corresponding feeding days. FCR was calculated as the ratio of total feed intake to total weight gain. Finally, RFI was calculated following the formula (Do et al., 2013).

$$\mathrm{RFI}=\mathrm{ADFI}-\left(\beta_{0}+\beta_{1}\,\mathrm{BW}+\beta_{2}*\mathrm{ADG}+\text{e}\right)$$

where β_0_ is the intercept, β_1_ represents the partial regression coefficient of ADFI on BW, β_2_ is the partial regression coefficient of ADFI on ADG, and e is the residual error.

The phenotype values of four FE related traits were calculated, and their corresponding descriptive statistics were performed (Table 1). The average FCR was 2.47 with a standard deviation of 0.52, whereas ADG and ADFI were 0.79 and 1.93 kg per day on average with a standard deviation of 0.12 and 0.38, respectively. RFI ranged from -1.00 to 0.83 kg with an average of 0.01 and a standard deviation of 0.32. The Student’s *t*-test showed that the data from the two batches of pigs were significantly different for FCR, ADFI, and RFI. The genetic correlations of FE related traits were calculated using ASREML software (Gilmour et al., 2015) four traits were analyzed together. The model fitted for FCR, ADG, ADFI, and RFI was:

**TABLE 1:** The descriptive statistics of four feed efficiency traits.

**Trait^a^**	**N-1^b^**	**N-2^c^**	**Mean**	**SD^d^**	**Min**	**Max**	**p_value^e^**
FCR	156	140	2.47	0.52	1.05	4.75	1.02E-09
ADG (kg/day)	156	140	0.79	0.12	0.38	1.18	0.89
ADFI (kg/day)	156	140	1.93	0.38	0.68	2.91	2.63E-10
RFI (kg)	156	140	0.01	0.32	–1.00	0.83	1.76E-14

*^*a*^FCR, feed conversion ratio; ADG, average daily gain; ADFI, average daily feed intake; RFI, residual feed intake. ^*b*^N-1 = number of individuals for each trait in the first batch. ^*c*^N-2 = number of individuals for each trait in the second batch. ^*d*^SD = standard deviation. ^*e*^p_value = p-value of Student’s test for two batches data at significance level 0.05.*

$$y=\mu +X\,\text{b}+Z_{1}\,a+Z_{2}\,\text{t}+e$$

with

$$E\,\left\{\begin{array}{c} \text{y}\\ \text{a}\\ \text{t}\\ \text{e} \end{array}\right\}=\left\{\begin{array}{c} \text{Xb}\\ 0\\ 0\\ 0 \end{array}\right\},\mathrm{Var}\,\left\{\begin{array}{c} \text{a}\\ \text{t}\\ \text{e} \end{array}\right\}=\left\{\begin{array}{ccc} \text{A}\,\sigma_{\text{a}}^{2}\; & 0\; & 0\\ 0\; & \text{I}\,\sigma_{\text{t}}^{2}\; & 0\\ 0\; & 0\; & \text{I}\,\sigma_{\text{e}}^{2} \end{array}\right\}$$

where, **y** is the vector of phenotypic values of four trait; **μ** is the population mean; **b** is the fixed effect of the batch; **a** is the vector of additive genetic effects; **t** is the vector of litter effects; **e** is a vector of residual error effects. **X**, **Z_1_**, and **Z_2_** are incidence matrices of **y** related to **b**, **a,** and **t**, respectively. **A** is the genetic relationship matrix, five generations of pedigree were traced back to construct A, and **σ_*a*_^2^** is the additive genetic variance. **I** is the identity matrix of appropriate dimension, **σ_*t*_^2^** is the variance of litter effect and **σ_*e*_^2^** is the residual variance.

Subsequently, genetic correlations were calculated based on the variance components as follows:

$$r_{A}=\frac{\mathrm{cov}\,\left(a_{1},a_{2}\right)}{\sigma_{a_{1}}\,\sigma_{a_{2}}}$$

where, r_*A*_ is the genetic correlation between trait 1 and trait 2, a_1_ and a_2_ represent the additive genetic values of trait 1 and trait 2 for same individuals, cov(a_1_,a_2_) and σ_*a1*_, σ_*a2*_ refer to the genetic covariance of two traits and genetic standard deviation of trait 1 and trait 2, respectively.

### Genotyping and Quality Control

Genomic DNA was extracted from ear samples using a TIANamp Blood DNA Kit (catalog number DP348; Tiangen, Beijing). Genotyping was performed on 50697 SNPs across the entire pig genome using a PorcineSNP50 BeadChip (Illumina, San Diego, CA, United States). Quality control was performed using PLINK 1.9 software (Chang et al., 2015). Individuals with call rates (CR) less than 95% were removed and then SNP with CR less than 95%, minor allele frequencies (MAF) < 5%, or significant deviation from the Hardy–Weinberg equilibrium (HWE; *P* < 10 × 10^–6^) were removed. After genotype quality control, no individuals were removed, and 41272 SNPs remained for further analysis.

### Genome-Wide Association Study

In this study, two different single-trait methods, single SNP linear model GWAS (LM-GWAS) and single-step GWAS (ssGWAS) were implemented to identify significant SNPs associated with FE related traits.

#### Linear Model GWAS (LM-GWAS)

A single SNP marker linear regression model was performed using the following single-trait animal model to detect the association of SNP with the four FE related traits, respectively. In order to control population stratification and to account for shared genetic effects of related individuals, a random polygenic effect was included in this model (Sanchez et al., 2014):

y=μ+batch+bx+g+e

where, **batch** is the fixed effect of the batch; **b** is the average effect of the gene substitution of a particular SNP; **x** is a vector of the SNP genotype (coded as 0, 1, or 2); **g** is a vector of random polygenic effects with a normal distribution **g**∼N(0, **G**σ^2^), in which σ^2^ is the additive polygenic variance, and **G** is the genomic relationship matrix constructed using all markers following Yang et al. (2011) **e** is a vector of residual effects with a normal distribution e∼N(0, **I**σ_*e*_^2^), where **I** is the identity matrix of appropriate dimension and σ_*e*_^2^ is the residual variance. For each SNP marker, the estimation of b and its sampling variance σ_*b*_^2^ can be obtained through the mixed model equations.

#### Single-Step GWAS (ssGWAS)

Compared to LM-GWAS, the following single-trait animal model in ssGWAS proposed by Wang et al. (2012) can simultaneously use all the SNP information:

y=Xb+Zu+e

where **b** is the fixed effect of batch; **u** is the vector of additive genetic effects with a normal distribution **μ**∼N(0, **G**σ_μ_^2^), σ_*u*_^2^ is the additive genetic variance, and **G** is same as in LM-GWAS. **X** and **Z** are incidence matrices of **y** related to **b** and **u**, respectively.

The effect of each SNP can be estimated by ssGWAS, following Aguilar et al. (2019) the *P*-value of each SNP was calculated:

$$p_{\text{i}}=P_{t}\,\left(\frac{{\text{û}}_{i}}{\sqrt{{\hat{\sigma }}_{i}^{2}/n}},n-1\right)$$

where P_*t*_ is the distribution function of t distribution, ${}_{i}^{u}$ is ith SNP effect, $\hat{\sigma }$_*i*_^2^ is the genetic variance of ith SNP, n is the number of animals with ith SNP.

The software GCTA (Yang et al., 2011) was used for the LM-GWAS method. The genetic variance of each SNP was also provided. For ssGWAS, blupf90 was to estimate genomic breeding values that were used to further estimate SNP effects and *P*-values via postGSf90 (Aguilar et al., 2018).

In order to control false positives, the False Discovery Rate (FDR) method for multiple testing was used (Benjamin and Hochberg, 1995; Weller et al., 1998). FDR was calculated as:

$$\mathrm{FDR}=\frac{\text{m}\times P_{M\,\text{ax}}}{n}$$

where **m** is the number of times to be tested, **n** is the number of significant SNPs at assigned FDR level, e.g., 0.01. **P_Max_** is the genome-wide significance level empirical *P*-value of FDR adjusted. Based on the *P*-values of SNPs obtained by two different methods, the empirical *P*-value of FDR adjusted at the genome-wide significance level of 0.01 was calculated on each trait for two methods in this study.

### Population Stratification

In order to assess the influence of population stratification on the GWAS, A quantile-quantile (Q-Q) plot was generated using PLINK 1.9 software.

### Identification of Candidate Genes

After identifying significant SNPs by GWAS, the genes located in or overlapping between the 0.25 Mb downstream and 0.25 Mb upstream region of the significant SNPs were determined using the Ensembl (Sus scrofa 11.1 genome version).^2^ QTLdb^3^ was used to annotate significant SNPs located in previously mapped QTLs in pigs. To identify the related pathways and function annotation, KEGG^4^ and Gene Ontology analyses^5^ were performed.

## Results

### Genetic Correlations of FE Related Traits

The genetic correlations of FE related traits ranged from 0.12 to 0.90, while the standard errors of all genetic correlations were high. Among the four FE related traits, FCR had the highest genetic correlations of 0.90 with ADFI, while it had the lowest genetic correlation of 0.12 with ADG. The corresponding standard errors were 0.17 and 0.59. The genetic correlation between FCR and RFI was 0.72 with standard error of 0.18. Similarly, the genetic correlation of RFI with ADFI was 0.71 with standard error of 0.14. The genetic correlations of ADG with ADFI and RFI were 0.51 and 0.38 with standard errors of 0.34 and 0.44, respectively.

### Population Stratification

**FIGURE 1:**
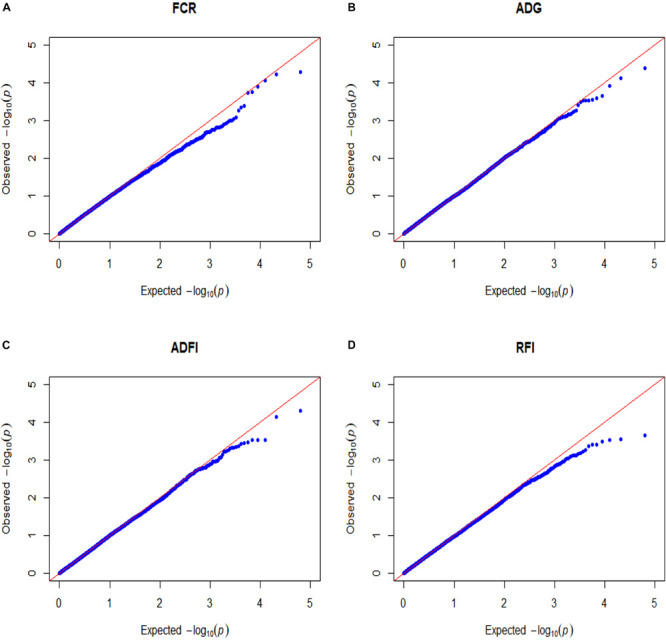
Quantile–quantile plots of GWAS for four feed efficiency traits in a Landrace population. FCR, Feed conversion ratio; ADG, Average daily gain; ADFI, Average daily feed intake; RFI, Residual feed intake. The *x*-axis and the *y*-axis represent the expected and observed -log_10_(*P*-value). **(A)** FCR. **(B)** ADG. **(C)** ADFI. **(D)** RFI.

False-positive results for significant SNPs are the most critical problem in GWAS. Therefore, it is essential to control population stratification and reduce the occurrence of false-positive results. The quantile-quantile plots (Q-Q plots) show that the influence of population stratification was negligible (Figure 1). Moreover, the average genomic inflation factors (λ) for the four FE related traits were close to 1 (range 1.02–1.09). The QQ plots and λ suggest that there were little or no residual population structure effects on the test statistic inflation. Despite the small sample size, the results of GWAS were reasonable and worth further investigation.

### Identification of Significant SNPs and QTL Regions Associated With FE Related Traits

**FIGURE 2:**
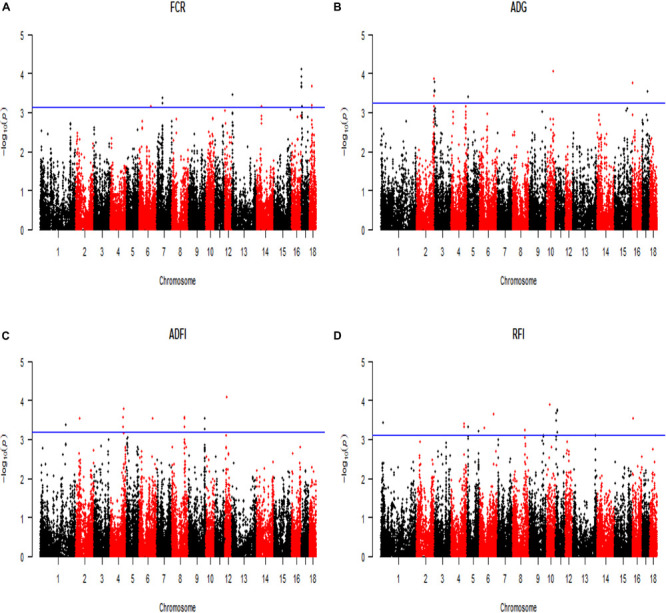
Manhattan plot of the genome-wide association analysis on four feed efficiency traits by using linear model GWAS (LM-GWAS) method. FCR, Feed conversion ratio; ADG, Average daily gain; ADFI, Average daily feed intake; RFI, Residual feed intake. In the Manhattan plots, negative log10 *P-*values of the quantified SNPs were plotted against their genomic positions. The *x*-axis and the *y*-axis represent the number of chromosome and the observed -log_10_(*P*-value), respectively. Different colors indicate various chromosomes. The blue lines indicate the genome-wide significant thresholds of FDR adjusted, respectively. For **(A)** FCR, it was 7.48 × 10^–4^. Similarly, **(B)** ADG was 5.64 × 10^–4^, **(C)** ADFI was 6.53 × 10^–4^, and **(D)** RFI was 7.58 × 10^–4^.

**FIGURE 3:**
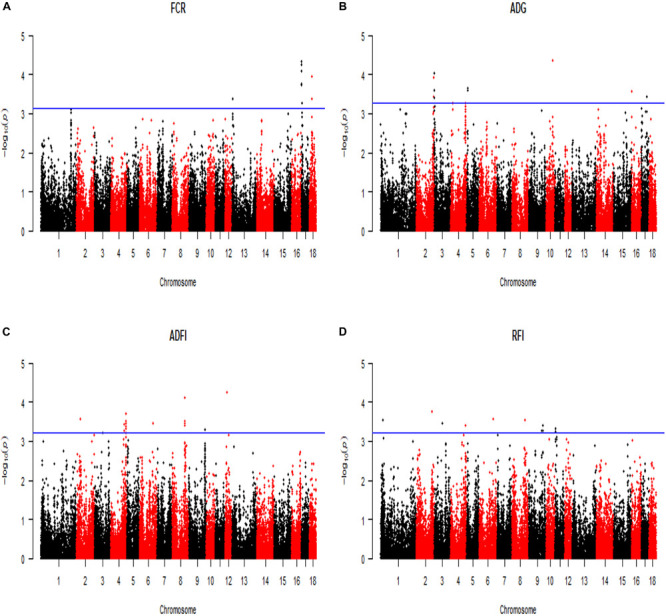
Manhattan plot of the genome-wide association analysis on four feed efficiency traits by using single-step GWAS (ssGWAS) method. FCR, Feed conversion ratio; ADG, Average daily gain; ADFI, Average daily feed intake; RFI, Residual feed intake. In the Manhattan plots, negative log10 *P-*values of the quantified SNPs were plotted against their genomic positions. The *x*-axis and the *y*-axis represent the number of chromosome and the observed -log_10_(*P*-value), respectively. Different colors indicate various chromosomes. The blue lines indicate the genome-wide significant thresholds of FDR adjusted, respectively. For **(A)** FCR, it was 7.17 × 10^–4^. Similarly, **(B)** ADG was 5.52 × 10^–4^, **(C)** ADFI was 6.16 × 10^–4^, and **(D)** RFI was 5.89 × 10^–4^.

**FIGURE 4:**
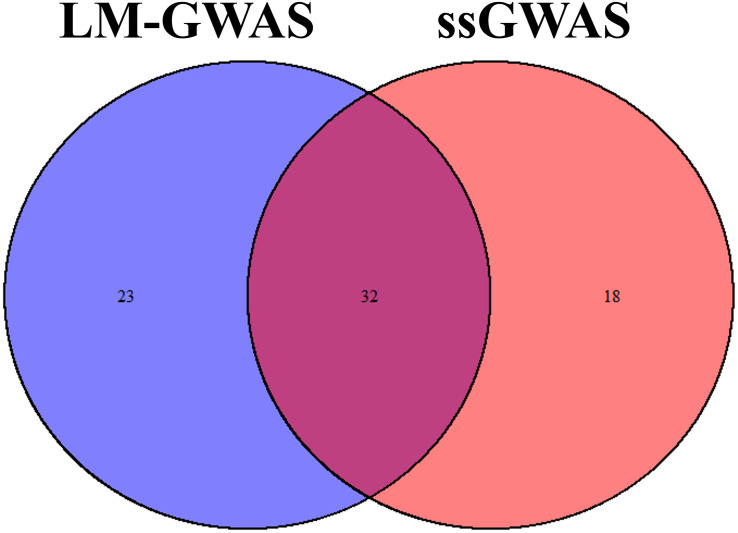
Venn plot of significant SNPs in two methods. Significant SNPs by linear model GWAS (LM-GWAS) and single-step GWAS (ssGWAS) for four traits.

All significant SNPs associated with the four FE related traits (FCR, ADG, ADFI, and RFI) identified by GWAS are illustrated in Supplementary Tables 1, 2 and Figures 2, 3. In LM-GWAS and ssGWAS methods, the empirical *P*-values of a multiple testing based on FDR adjusted at the genome-wide significance level of 0.01 for FCR were 7.48 × 10^–4^ and 7.17 × 10^–4^, respectively. For ADG, ADFI and RFI, the genome-wide empirical *P*-values obtained by LM-GWAS were 5.64 × 10^–4^, 6.53 × 10^–4^ and 7.58 × 10^–4^, and ssGWAS were 5.24 × 10^–4^, 6.16 × 10^–4^ and 5.89 × 10^–4^, respectively. A total of 55 and 50 genome-wide significant SNPs were found by LM-GWAS and ssGWAS methods, 32 SNPs out of them were common (Figure 4). Among the 55 significant SNPs identified by the LM-GWAS method, 15, 11, 13, and 16 SNPs were related to FCR, ADG, ADFI, and RFI, and correspondingly explained 2.66, 1.33, 1.64, and 1.80% additive genetic variance, respectively. These SNPs were mainly located on all autosomes except SSC15 (Supplementary Table 1). Among them, two significant SNPs (WU_10.2_6_122065838, WU_10.2_4_116973174) were associated with both ADFI and RFI. The ssGWAS method identified 9, 13, 17, and 11 significant SNPs associated with FCR, ADG, ADFI and RFI, which were mainly located on SSC3, 4, 8, 9, and 17, respectively (Supplementary Table 2), and explained 1.20, 1.79, 2.07, and 1.29% additive genetic variance. Among the 50 SNPs, three common SNPs (WU_10.2_6_122065838, ALGA0049005 and ALGA0019602) were all significant in both ADFI and RFI. In addition, the SNP WU_10.2_6_122065838 was also identified in the LM-GWAS method.

Meanwhile, 13 regions were identified by two methods, as shown in Table 2. Among them, eight common regions were found by two different methods. Three regions for FCR were found located on SSC7, SSC17, and SSC18, respectively. Four regions for ADG were identified on SSC2, SSC3, SSC4, and SSC5. Four regions for ADFI and five regions for RFI were also identified. Among them, one region for ADG and ADFI, and two regions for ADFI and RFI overlapped. According to Pig QTLdb,^1^ eight regions identified in our study overlapped or were close to the reported QTLs related to FCR, ADG, ADFI, and RFI. Among them, four regions overlapped and four regions were nearby the reported QTLs.

**TABLE 2:** Quantitative traits loci (QTLs) related to four feed efficiency traits.

**Trait^a^**	**Method^b^**	**Chromosome**	**Region (Mb)^c^**	**N^d^**	**QTL region (Mb)^e^**	**Traits related with QTL**
FCR	a	7	**45.83–45.86**	2	45.10–53.98	Average daily gain (Quintanilla et al., 2002)
	a&b	17	1.15–2.69	7	0.44–20.053	Body weight (Guo Y. M. et al., 2008b)
	a&b	18	20.48–20.96	2	21.47–21.47	Average daily gain (Wang et al., 2015)
ADG	a&b	2	147.07–147.11	2	148.27–148.27	Body depth/width (Fan et al., 2009)
	a&b	3	0.35–0.54	3	1.79–4.20	Body weight (Fan et al., 2009)
	a&b	5	8.85–8.87	3	9.29–9.29	Average daily gain (Rothammer et al., 2014)
ADG\ADFI	b	4	120.99–121.96	5	120.51–134.90	Body weight (Bartenschlager and Geldermann, 2003)
ADFI	a&b	9	**128.77–129.09**	2	128.00–128.99	Daily feed intake (Reyer et al., 2017)
					128.99–147.51	Body weight (10 weeks) (Yoo et al., 2014)
ADFI\RFI	a&b	4	**105.57–106.83**	6	105.64–105.64	Feed conversion ratio (Wang et al., 2015)
					106.51–124.31	Average daily gain (Malek et al., 2001)
	a&b	8	102.88–105.56	6	107.04–113.33	Feed intake per feeding (Ding et al., 2017)
RFI	b	9	112.25–112.31	3	114.32–123.52	Lipid accretion rate (Duthie et al., 2008)
	a	11	**5.38–6.37**	2	6.01–6.99	Residual feed intake (Jiao et al., 2014)
			16.03–16.08	3	15.85–15.85	Feed conversion ratio (Horodyska et al., 2017)

*^*a*^FCR, Feed conversion ratio; ADG, Average daily gain; ADFI, Average daily feed intake; RFI, Residual feed intake. ^*b*^a = linear model GWAS (LM-GWAS), b = single-step GWAS (ssGWAS). ^*c*^Region (Mb) = the merging of neighbored significant SNPs. ^*d*^N = Number of significant SNPs in the region. ^*e*^QTL region (Mb) = The QTL regions reported in Pig QTLdb. Values in bold had an overlap region with QTLs for FE related traits in Pig QTLdb.*

### Identification of Candidate Genes

All the significant SNPs identified by the two methods were annotated within the 0.25 Mb downstream and upstream region with reference to the Sus scrofa 11.1 genome assembly. GO analysis separately revealed 12 candidate genes for LM-GWAS and 7 candidate genes for ssGWAS (Table 3). Combined these two methods, 13 positional candidate genes were detected for the four FE related traits. Among them, six genes had function related to FCR, two genes for ADG, four genes for ADFI, and two for RFI. These 13 candidate genes have a highlight biology function with FE, which involved in biological processes such as lipid metabolism, carbohydrate metabolism, lipid transport, and regulation of insulin secretion. Among them, six genes were identified in both LM-GWAS and ssGWAS methods.

**TABLE 3:** Significant SNPs and related genes for four feed efficiency traits.

**Trait^a^**	**Method^b^**	**SNP name**	**Chromosome**	**Position (bp)**	**Gene^c^**	**Distance^d^**	**Gene function**
FCR	a&b	WU_10.2_13_3842462	13	3,655,526	*PLCL2*	-150,373	Lipid catabolic process
	a	ASGA0062927	14	40,507,969	*SIRT4*	+204,081	Negative regulation of insulin secretion
					*PLA2G1B*	+185,650	Lipid metabolic process
	a	ASGA0062929	14	40,548,521	*PLA2G1B*	+226,202	Lipid metabolic process
	a	ALGA0097485	18	23,538,749	*SPAM1*	-51,203	Carbohydrate metabolic process
					*HYAL4*	-85,477	
					*ENSSSCG00000016602*	-88,233	
ADG	a&b	ASGA0100941	2	147,068,762	*PRELID2*	-40,819	Phospholipid transport process
	a&b	WU_10.2_2_153522747	2	147,112,980	*PRELID2*	Within	Phospholipid transport process
	a&b	WU_10.2_3_329436	3	537,968	*GPER1*	-174,192	Positive regulation of insulin secretion; negative regulation of lipid biosynthetic process
ADFI	a	ASGA0005581	1	203,367,107	*ACER2*	-19,714	Lipid metabolic process
	b	ALGA0019602	3	68,298,863	*HK2*	+67,248	Glucose metabolic process; glycolytic process
	a&b	ALGA0065251	12	14,839,808	*TEX2*	Within	Lipid transport process
					*ICAM2*	-42115	Insulin metabolic process
RFI	b	ALGA0019602	3	68,298,863	*HK2*	+67,248	Glucose metabolic process; glycolytic process
	b	WU_10.2_11_4727497	11	50,92,611	*PDX1*	-211,028	Glucose metabolic process; insulin secretion
	a&b	ALGA0060467	11	53,79,554	*PDX1*	+68,711	Glucose metabolic process; insulin secretion

*^*a*^FCR, Feed conversion ratio; ADG, Average daily gain; ADFI, Average daily feed intake; RFI, Residual feed intake. ^*b*^a, linear model GWAS (LM-GWAS); b, single-step GWAS (ssGWAS). ^*c*^PLCL2, Phospholipase C Like 2; SIRT4, Sirtuin 4; PLA2G1B, Phospholipase A2 Group IB; SPAM1, Sperm Adhesion Molecule 1; HYAL4, Hyaluronidase 4; PRELID2, PRELI Domain Containing 2; GPER1, G Protein-coupled Estrogen Receptor 1; ACER2, Alkaline Ceramidase 2; HK2, Hexokinase 2; TEX2, Testis Expressed 2; ICAM2, Hexokinase 2; PDX1, Pancreatic and Duodenal Homeobox 1. ^*d*^+/-: The SNP located in the upstream/downstream region of the nearest gene.*

## Discussion

### Sample Size for GWAS

Sample size is a key factor for the efficiency of GWAS; one drawback of this study is that only 296 Landrace pigs were used to detect the genetic variants related to FE related traits. Compared to other traits, the measurements of FE related traits are usually difficult. Besides, it is not easy to acquire a large sample size. For instance, Ding et al. (2017) used a comparable population size of 338 Duroc boars to detect feeding behavior and eating efficiency by GWAS. Ramayo-Caldas et al. (2019) integrated GWAS and gene expression to identify putative regulators and predictors of FE using 350 Duroc pigs. To minimize the effect of the small sample size on GWAS, the phenotypes were strictly measured in this study, and two different methods were implemented, which adopted a single-marker only or multiple SNPs simultaneously to take into account the genetic correlations among relevant traits.

### Genetic Correlations and Significant SNPs With Pleiotropy

GWAS performed in pigs revealed significant associations for economically-relevant traits. In recent years, researches performed GWAS on FE related traits, unveiling high genetic correlations. For instance, Godinho et al. (2018) found that in purebred pig ADFI highly genetically correlates with FCR (0.71) and RFI (0.73), respectively. FCR had a high correlation with RFI (0.82). Do et al. (2013) also found high genetic correlations between FE related traits in Landrace. They found the genetic correlation of ADG and ADFI was 0.72. RFI had highly genetic correlations with FCR (0.91) and ADFI (0.84). Similar results were also found in our study (Table 2). Furthermore, our results showed that some significant SNPs with pleiotropic effects were identified. Four SNPs were significantly associated with both ADFI and RFI. Moreover, significant SNPs identified in GWAS overlap between some extent for these two traits. Based on the regions of merging neighbored significant SNPs, significant SNPs for ADFI (4 SNPs) and RFI (2 SNPs) were located in a region of 105.57–106.83 Mb on SSC4. Similarly, the regions of 102.88–105.56 Mb on SSC8 correlated with both RFI and ADFI. We speculate that due to the pleiotropy of the SNPs associated with one trait (ADFI/RFI), these traits tend to affect multiple additional phenotypes. Likewise, in cattle, many concordant QTLs between RFIp (calculated from linear phenotypic regression) and RFIg (calculated from linear genetic regression) were reported by Nkrumah et al. (2007) 14 common and 3 distinct QTLs were identified for the two RFI measures.

### The Comparison of Different Methods

Single SNP regression model is widely used in GWAS to identify the association of SNP with traits of interest, whereas it usually yields a high false-positive rate due to ignoring the linkage disequilibrium between adjacent SNPs. Some researchers investigated a haplotype-based sliding window strategy to reduce the false-positive by using multiple SNPs simultaneously. Some studies indicated that sliding window could result in different QTL regions, genes and SNPs with a single SNP method (Guo Y. et al., 2008; Braz et al., 2019) while some results showed that sliding window for GWAS could be complementary to single SNP analysis (Lorenz et al., 2010; Guerra et al., 2019). The controversy may perhaps depend on the different genetic architecture of the target trait. Recently, ssGWAS, which enables simultaneous analysis of an extensive array of SNPs, demonstrated its superiority in the reduction of false-positive rates (Wang et al., 2012). Therefore, ssGWAS and LM-GWAS were both used in our study to reduce false-positive rates and identified the correctness of our results. Our results reinforce the high consistency of these two methods, in which 32 common significant SNPs were identified by both methods (Figure 4). Meanwhile, Wang et al. (2014) reported that ssGWAS and a single-marker model had similar results in broiler chickens. So all these two methods could improve the power of GWAS and the accuracy of significant SNPs selected.

### QTLs Related to FE Related Traits

Alignment of the genetic and physical maps on the Sus scrofa 11.1 genome assembly (Pig QTLdatabase) enabled comparison of the QTLs detected in our study with previously described QTL regions, and several of QTLs were selected for further analysis. The most prominent common regions for FCR were identified on SSC17, and 18. A QTL from this study located at 20.48–20.96 Mb on SSC18 coincides with a QTL identified for ADG in a Duroc sire population mapped across the region of 21.47–21.47 Mb (Wang et al., 2015). For ADFI, the most promising QTLs were detected on porcine SSC9. A QTL located at 128.77–129.09 Mb on SSC9 overlapped with a QTL for ADFI in a pig line (Reyer et al., 2017). In addition, other QTLs located in 1.15–2.69 Mb (SSC17) for FCR and 0.35–0.54 Mb (SSC3) for ADG were all also consistent with QTLs for BW (Guo Y. M. et al., 2008; Fan et al., 2009). Two interesting regions containing all 6 significant SNPs for both ADFI and RFI were detected in the regions of 105.57–106.83 Mb on SSC4 and 102.88–105.56 Mb on SSC8. Among them, the QTL located in 105.57–106.83 Mb overlapped with two identified QTLs for FCR in the regions of 105.64–105.64 Mb and 106.51–124.31 Mb (Wang et al., 2015). Thus, this QTL has been independently discovered in different populations for ADFI/RFI and FCR, which further supports the biological relevance of common genetic variation on FE related traits (Becker et al., 2013). Another QTL for ADFI/RFI traits located at 102.88–105.56 Mb on SSC8 found in this study was in close proximity to a QTL (107.04–113.33 Mb) for ADG reported by Haggman and Uimari (2017). The remaining QTL regions for ADG identified in this study on SSC2 and 5 were close to regions affecting FE related traits and growth rate according to literature reports (Fan et al., 2009; Rothammer et al., 2014). Gregersen et al. (2012) reported a limited overlap of QTL for a particular trait between breeds. Although the sample size was limited, our result also had a large number of overlapping and coinciding QTL regions is in accordance with others’ GWAS results, and it suggests that our present study is reliable and accurate in a certain extent, and worth of further research for verifying candidate genes.

### Potential Candidate Genes

#### Potential Candidate Genes for FCR

Growth rate and feed intake were major influencing factors of FCR. One candidate gene Phospholipase A2 Group IB (*PLA2G1B*), which can encode a secreted member of the phospholipase A2 (*PLA2*), is crucial for the biological functions of lipid metabolic and catabolic processes. Hollie and Hui (2011) found that *PLA2G1B* affects the inhibition of lysophospholipid absorption, and limits lipid catabolic process. Additionally, *PLA2G1B* also produces lysophospholipids that limit hepatic fat catabolism and reduce energy consumption (Labonte et al., 2010). In previous studies on pigs, lipid metabolism pathway and energy pathway closely associated with RFI in muscle and adipose tissues (Lkhagvadorj et al., 2010; Vincent et al., 2015; Gondret et al., 2016). The *PLA2G1B* gene can have an effect on lipid metabolism in pigs and thus, on FCR, the ratio between feed intake and BW gain. Another candidate gene, Sirtuin 4 (*SIRT4*), has a vital role in glutamine metabolism and negative regulation of insulin secretion. Some researchers found that this gene is a regulator of insulin secretion, and it can reduce pancreatic insulin secretion (Anderson et al., 2017; Zaganjor et al., 2017; Huynh et al., 2018). Insulin sensitivity modulation and glucose handling influence energy metabolism and FE related traits (Fontanesi et al., 2012). Moreover, Do et al. (2014b) reported that insulin secretion affects the metabolic process of carbonization, which drives feed intake and FCR. In a previous study, lower insulin secretion led to a decrease in RFI, which triggered fat deposition (Hoque et al., 2009). The other four candidate genes (*PLCL2, phospholipase C like 2; SPAM1, Sus scrofa sperm adhesion molecule 1; HYAL4, hyaluronidase 4; ENSSSCG00000016602*) which were reportedly related to FE traits in pigs, are similarly involved in fat synthesis and decomposition processes, as well as insulin and lipid metabolism, and lipid transport.

#### Potential Candidate Genes for ADG

An important FE related trait is ADG, and many animal nutritionists consider this trait to be a major ethological concern. The most significant locus, *ASGA0036538*, was closest to the G Protein-coupled Estrogen Receptor 1 (*GPER1*) gene. *GPER1* is associated with positive regulation of insulin secretion, inhibition of fat cell differentiation (Williams, 2012) and insulin signal pathway (Kumar et al., 2011). Hayes et al. (2011) reported that the *GPER1* gene could stimulate the release of insulin and prevent the apoptosis of pancreatic beta cells. Although GPER1 has not been reported in pigs, it was associated with activate estrogen receptors involved in the hypothalamic control of multiple homeostatic functions in mice, such as energy metabolism (Hadjimarkou and Vasudevan, 2018). Do et al. (2014a) found that genes which are involved in insulin signaling and energy metabolism pathway played an important function in the regulation of FE related traits, such as RFI. Our study confirmed previous investigations. As to PRELI Domain Containing 2 (*PRELID2*), which is associated with phospholipid transport process and related pathway. In previous GWAS studies on pigs, the *PRELID2* gene has been reported to be associated with reproduction traits in Yorkshire pigs (Wang et al., 2018). Its relation to ADG, to the best of our knowledge, this genetic association was reported in pigs for the first time. Auqui et al. (2019) found that fatter pigs had higher cholesterol levels, suggesting a link between cholesterol levels and body weight. Their results demonstrated that *PRELID2* could significantly regulate body weight through cholesterol levels. The three genes above were found by both LM-GWAS and ssGWAS methods.

#### Potential Candidate Genes for ADFI

Some breeders are concerned about ADFI because this trait is highly associated with FE and growth rate (Do et al., 2013). Both LM-GWAS and ssGWAS methods identified a significant locus, ALGA0065251. According to the annotation, Intercellular adhesion molecule 2 (*ICAM2*) and Testis expressed 2 (*TEX2*) are associated with ADFI. *ICAM2* has a key function in glucose stimulus and insulin metabolic process (Qiu et al., 2008). The relation between insulin signaling pathway and RFI has been shown in pigs (Do et al., 2014b) and cattle (Chen et al., 2012; Rolf et al., 2012). In pigs, *ICAM2* gene is reported a meat quality traits involved in lipid metabolism and intramuscular fat deposition (Jeong et al., 2015). Researchers suggested that *ICAM2* was closely related to glucose stimulus and insulin metabolic processes, which are important for FE. Grefhorst et al. (2019) reported that *TEX2* promotes the transfer of cholesterol and other lipids in different cyclic lipoprotein. Our results show that *TEX2* is associated with the ADFI trait in Landrace pigs for the first time. Of note, a strong association between *TEX2* and FE, and growth rate in broilers was reported (Willson et al., 2017). Another candidate gene Alkaline Ceramidase 2 (*ACER2*), is closely associated with lipid metabolism (Zhang et al., 2019). This gene was identified by the LM-GWAS method. Several studies also support the notion that lipid metabolism is associated with RFI in pigs (Lkhagvadorj et al., 2010) and cattle (Herd and Arthur, 2009). Lkhagvadorj et al. (2010) found many genes in fat and liver that were differently expressed in low and high RFI pigs in response to caloric restriction and indicated that lipid metabolic pathways were important for regulation of RFI. Nevertheless, the lipid metabolic process is a very broad term, and therefore it might be worthy of further investigation to identify the exact sub-process that are involved in ADFI metabolism. Besides, one candidate gene (*HK2, Hexokinase 2*) was identified by the ssGWAS method. This gene was reportedly associated with glucose metabolism, insulin secretion, and glycolytic regulation (Wei et al., 2016; Miller et al., 2019). Our findings support a better understanding of the ADFI trait in pig lipid metabolism.

#### Potential Candidate Genes for RFI

RFI is an essential trait for animal husbandry and many studies have been conducted to investigate the genetic architecture underlying this trait. A variety of pathways may mediate RFI, such as hormones and growth factors that act through receptor tyrosine kinases [e.g., epidermal growth factor (EGF), insulin] (Hayes et al., 2011). Only one common locus *ALGA0060467* was found significantly associated with RFI in the current study and it is located the upstream of the Pancreatic Duodenal Homeobox 1 (*PDX1*). SNP, WU_10.2_11_4727497, associated with RFI was identified by ssGWAS method, it is also close to PDX1. Notably, the direct link between *PDX1* and FE related traits has not been reported previously in pigs. Interestingly, *PDX1* is associated with some classical pathways such as glucose/energy metabolism and insulin secretion pathway. In the adult endocrine pancreas, *PDX1* is a pivotal factor for the up-regulation of insulin gene transcription that, in turn, regulates somatostatin, the expression of glucokinase, glucose transporter protein-2, and islet amyloid peptide (Park et al., 2008). Do et al. (2014b) reported that insulin signaling pathway plays important roles in controlling RFI in Duroc pigs, and it has been shown that insulin also affects feed intake and feed behavior in chickens (Shiraishi et al., 2008, 2011). Moreover, many reports showed that *PDX1* was closely related to porcine pancreas development (Choi et al., 2009) and diabetes (Matsunari et al., 2014). Pancreas and diabetes are closely related to feed intake, digestion, absorption, and metabolism. Hence, *PDX1* might exert a vital function on feed intake and feed behavior. Another gene *HK2*, was found significantly associated with both RFI and ADFI by using the ssGWAS method as mentioned before.

## Conclusion

In summary, the present study indicated that the result of LM-GWAS and ssGWAS methods are highly consistent. Combining LM-GWAS and ssGWAS improved not only the power of GWAS in a small population but also allowed screening of candidate genes with high reliability (such as *PLA2G1B* and *PRELID2)*. This study provides a better understanding of the genetic mechanisms underlying feed efficiency related traits, which offers an opportunity for increased feed efficiency using marker-assisted selection or genomic selection in pigs.

## Data Availability Statement

The SNP array dataset generated for this study can be found in Figshare https://figshare.com/articles/GWAS/12431975.

## Ethics Statement

The animal study was reviewed and approved by the Institutional Animal Care and Use Committee (IACUC) at the China Agricultural University (permit number DK996). Written informed consent was obtained from the owners for the participation of their animals in this study.

## Author Contributions

XD designed and supervised the study. LF and YaJ conducted the statistical analyses and interpretation of results. MM, ZZ, and WC conceived the experimental traits. YiJ and HS prepared the samples for DNA genotyping. LF and YaJ wrote the manuscript, which was critically remarked by XD. All authors contributed to the article and approved the submitted version.

## Conflict of Interest

The authors declare that the research was conducted in the absence of any commercial or financial relationships that could be construed as a potential conflict of interest.

## Footnotes

1 http://www.animalgenome.org/cgi-bin/QTLdb/SS/index

2 http://www.ensembl.org/Sus_scrofa/Info/Index/

3 http://www.animalgenome.org/cgi-bin/QTLdb/SS/download?file=gbpSS_11.1

4 http://www.kegg.jp/kegg/pathway.html/

5 http://www.pantherdb.org/
