# Supplementary material for: A Genome-Wide Association Study on Feed Efficiency Related Traits in Landrace Pigs
Source: Front Genet. 2020 Jul 3;11:692. doi: 10.3389/fgene.2020.00692 (PMC7350416; doi:10.3389/fgene.2020.00692)
Supplement: Supplementary file 1 [file Data_Sheet_1.pdf]

**Supplementary Table 1. Significant SNPs and associated genes for four traits in GWAS by using linear model GWAS (LM-GWAS) method**

| Trait[1] | $P_{(empirical)}$_value[2] | SNP name | Chromosome | Position (bp) | P_value | Gene | Gene function |
|---|---|---|---|---|---|---|---|
| FCR | 7.48E-04 | **WU_10.2_17_1001798** | 17 | 1152272 | 7.80E-05 | *TRMT9B* | oxidation-reduction process |
| | | **ALGA0092581** | 17 | 1703513 | 1.17E-04 | *ENSSSCG00000006973* | cell-cell junction assembly |
| | | **WU_10.2_17_1851007** | 17 | 1956407 | 1.70E-04 | *SGCZ* | NA |
| | | **DIAS0000567** | 18 | 20961153 | 2.05E-04 | *GRM8* | signal transduction |
| | | **WU_10.2_17_1434901** | 17 | 1636763 | 2.13E-04 | *C8orf48* | NA |
| | | **WU_10.2_17_1697296** | 17 | 1935638 | 2.23E-04 | *SGCZ* | NA |
| | | **WU_10.2_13_3842462** | 13 | 3655526 | 3.51E-04 | ***PLCL2*** | lipid catabolic process; lipid catabolic process |
| | | ASGA0033576 | 7 | 45856658 | 4.11E-04 | *PKHD1* | cellular calcium ion homeostasis |
| | | ALGA0041464 | 7 | 45828581 | 5.62E-04 | | |
| | | **MARC0055137** | 18 | 20484602 | 6.31E-04 | *SND1* | miRNA catabolic process |
| | | WU_10.2_17_2202762 | 17 | 2346395 | 6.80E-04 | *ENSSSCG00000039907* | NA |
| | | ALGA0077090 | 6 | 39608538 | 6.82E-04 | *ENSSSCG00000015023* | mannosylation |
| | | ASGA0062927 | 14 | 40507969 | 6.82E-04 | ***SIRT4*** | negative regulation of fatty acid oxidation; negative regulation of insulin secretion; tricarboxylic acid metabolic process; positive regulation of lipid biosynthetic process |
| | | | | | | ***PLA2G1B*** | lipid metabolic process; fatty acid biosynthetic process; phospholipid metabolic process; lipid catabolic process |
| | | ASGA0062929 | 14 | 40548521 | 7.08E-04 | ***PLA2G1B*** | lipid metabolic process; fatty acid biosynthetic process; phospholipid metabolic process; lipid catabolic process |

| | | | | | | | |
|---|---|---|---|---|---|---|---|
| | | ALGA0097485 | 18 | 23538749 | 7.33E-04 | ***SPAM1***<br>***HYAL4***<br>***ENSSSCG00000016602*** | carbohydrate metabolic process |
| ADG | 5.64E-04 | **WU_10.2_10_59997776** | 10 | 54208829 | 8.47E-05 | *PLXDC2* | NA |
| | | **ASGA0100941** | 2 | 147068762 | 1.37E-04 | ***PRELID2*** | phospholipid transport process |
| | | **WU_10.2_3_198486** | 3 | 350694 | 1.62E-04 | *PRKAR1B* | cGMP-mediated signaling |
| | | **H3GA0054878** | 16 | 605799 | 1.73E-04 | *CTNND2* | cell-cell adhesion |
| | | **WU_10.2_3_346336** | 3 | 521070 | 2.66E-04 | *SUN1* | nuclear matrix anchoring at nuclear membrane |
| | | **MARC0009678** | 17 | 44751346 | 2.82E-04 | *ENSSSCG00000038990* | peptidyl-tyrosine dephosphorylation |
| | | **WU_10.2_3_329436** | 3 | 537968 | 2.89E-04 | ***GPER1*** | positive regulation of insulin secretion;<br>negative regulation of lipid biosynthetic process |
| | | **WU_10.2_2_153522747** | 2 | 147112980 | 3.75E-04 | ***PRELID2*** | phospholipid transport process |
| | | **5_6139359** | 5 | 8845224 | 3.99E-04 | | |
| | | **ALGA0030157** | 5 | 8854690 | 3.99E-04 | *TAB1* | activation of protein kinase activity |
| | | **WU_10.2_5_6163751** | 5 | 8869294 | 3.99E-04 | | |
| ADFI | 6.53E-04 | **ALGA0065251** | 12 | 14839808 | 8.11E-05 | ***TEX2***<br>***ICAM2*** | lipid transport process<br>insulin metabolic process |
| | | **WU_10.2_4_116973174** | 4 | 106826499 | 1.63E-04 | *PHTF1* | signal transduction |
| | | **ASGA0039519** | 8 | 105054796 | 2.67E-04 | *METTL14* | forebrain radial glial cell differentiation |
| | | **ALGA0049005** | 8 | 105105852 | 2.72E-04 | *PRSS12* | zymogen activation |
| | | WU_10.2_4_116040517 | 4 | 105851764 | 2.76E-04 | *NRAS* | signal transduction |
| | | **ASGA0100809** | 9 | 129091764 | 2.77E-04 | ENSSSCG00000015023 | mannosylation |
| | | **M1GA0012018** | 8 | 104924079 | 2.81E-04 | *SEC24D* | COPII-coated vesicle cargo loading |

| | | | | | | | |
|---|---|---|---|---|---|---|---|
| | | **ASGA0084451** | 2 | 31158784 | 2.86E-04 | *ENSSSCG00000034018* | translation |
| | | **WU_10.2_6_122065838** | 6 | 131840097 | 2.87E-04 | *ADGRL2* | cell surface receptor signaling pathway |
| | | ASGA0005581 | 1 | 203367107 | 4.05E-04 | ***ACER2*** | lipid metabolic process |
| | | **WU_10.2_8_113454144** | 8 | 105564831 | 4.83E-04 | *ENSSSCG00000009116* | Rab protein signal transduction |
| | | WU_10.2_4_115704336 | 4 | 105565718 | 4.87E-04 | *SYCP1* | regulation of protein localization |
| | | **WU_10.2_9_141498530** | 9 | 128772045 | 5.44E-04 | *KCNK2* | G protein-coupled receptor signaling pathway |
| RFI | 7.58E-04 | MARC0077909 | 10 | 40140575 | 1.28E-04 | *BAMBI* | cell migration |
| | | ASGA0050023 | 11 | 16074356 | 1.69E-04 | *ENSSSCG00000033483* | NA |
| | | M1GA0015002 | 11 | 16080206 | 1.89E-04 | | |
| | | **ALGA0060467** | 11 | 5379554 | 2.14E-04 | ***PDX1*** | glucose metabolic process; insulin secretion |
| | | **WU_10.2_6_122065838** | 6 | 131840097 | 2.21E-04 | *ADGRL2* | cell surface receptor signaling pathway |
| | | WU_10.2_16_7550558 | 16 | 7078401 | 2.80E-04 | *NSSSCG00000016796* | NA |
| | | H3GA0031138 | 11 | 6366025 | 3.34E-04 | *MTUS2* | NA |
| | | **WU_10.2_1_18721969** | 1 | 16417185 | 3.73E-04 | *PPIL4* | protein peptidyl-prolyl isomerization |
| | | WU_10.2_4_116973174 | 4 | 106826499 | 3.89E-04 | *PHTF1* | signal transduction |
| | | WU_10.2_4_116864084 | 4 | 106658685 | 4.72E-04 | *AP4B1* | vesicle-mediated transport |
| | | WU_10.2_5_8988909 | 5 | 11256612 | 4.75E-04 | *FOXRED2* | ubiquitin-dependent ERAD pathway |
| | | ALGA0037549 | 6 | 158658647 | 4.96E-04 | *GLIS1* | negative regulation of transcription by RNA polymerase II |
| | | ASGA0092485 | 8 | 102881678 | 5.63E-04 | *TNIP3* | cellular response to lipopolysaccharide |
| | | ALGA0048953 | 8 | 102999435 | 5.63E-04 | | |
| | | H3GA0017183 | 5 | 94558952 | 5.93E-04 | *C12orf50* | Poly (A)+ mRNA export from nucleus |
| | | H3GA0031439 | 11 | 16032696 | 6.28E-04 | *ENSSSCG00000033483* | NA |

[1]FCR = feed conversion ratio, ADG = average daily gain, ADFI = average daily feed intake, RFI = residual feed intake.

[2]$P_{(empirical)}$ _value = the empirical p-values of a multiple testing based on FDR adjusted at the genome-wide significance level of 0.01.

The bold data in SNP name column represents common SNPs identified by linear model GWAS (LM-GWAS) and single-step GWAS (ssGWAS) methods.

The underscore data in SNP name column represents common SNPs related to ADFI and RFI traits.

The bold data in Gene column represent genes related to four feed efficiency related traits.

**Supplementary Table 2. Significant SNPs and associated genes for four traits in GWAS by using single-step GWAS (ssGWAS) method**

| Trait[1] | $P_{(empirical)}$_value[2] | SNP name | Chromosome | Position (bp) | P_value | Gene | Gene function |
|---|---|---|---|---|---|---|---|
| FCR | 7.17E-04 | **WU_10.2_17_1001798** | 17 | 1152272 | 4.76E-05 | *TRMT9B* | oxidation-reduction process |
| | | **WU_10.2_17_1697296** | 17 | 1935638 | 5.65E-05 | *SGCZ* | NA |
| | | **ALGA0092581** | 17 | 1703513 | 8.11E-05 | *ENSSSCG00000006973* | cell-cell junction assembly |
| | | **DIAS0000567** | 18 | 20961153 | 1.15E-04 | *GRM8* | signal transduction |
| | | **WU_10.2_17_1434901** | 17 | 1636763 | 1.68E-04 | *C8orf48* | NA |
| | | **WU_10.2_17_1851007** | 17 | 1956407 | 1.80E-04 | *SGCZ* | NA |
| | | **MARC0055137** | 18 | 20484602 | 4.14E-04 | *SND1* | miRNA catabolic process |
| | | **WU_10.2_13_3842462** | 13 | 3655526 | 4.26E-04 | ***PLCL2*** | lipid catabolic process; lipid catabolic process |
| | | WU_10.2_17_2592359 | 17 | 2686699 | 5.36E-04 | *SGCZ* | NA |
| ADG | 5.24E-04 | **WU_10.2_10_59997776** | 10 | 54208829 | 4.28E-05 | *PLXDC2* | NA |
| | | **WU_10.2_3_198486** | 3 | 350694 | 9.29E-05 | *PRKAR1B* | cGMP-mediated signaling |
| | | **ASGA0100941** | 2 | 147068762 | 1.18E-04 | ***PRELID2*** | phospholipid transport process |
| | | **WU_10.2_5_6163751** | 5 | 8869294 | 2.18E-04 | *TAB1* | activation of protein kinase activity |
| | | **WU_10.2_3_329436** | 3 | 537968 | 2.46E-04 | ***GPER1*** | positive regulation of insulin secretion; negative regulation of lipid biosynthetic process |
| | | **5_6139359** | 5 | 8845224 | 2.54E-04 | *TAB1* | activation of protein kinase activity |
| | | **ALGA0030157** | 5 | 8854690 | 2.54E-04 | | |
| | | **H3GA0054878** | 16 | 605799 | 2.71E-04 | *CTNND2* | cell-cell adhesion |
| | | **WU_10.2_2_153522747** | 2 | 147112980 | 3.68E-04 | ***PRELID2*** | phospholipid transport process |
| | | **MARC0009678** | 17 | 44751346 | 3.75E-04 | *ENSSSCG00000038990* | peptidyl-tyrosine dephosphorylation |
| | | **WU_10.2_3_346336** | 3 | 521070 | 3.83E-04 | *SUN1* | nuclear matrix anchoring at nuclear membrane |

| | | | | | | | |
|---|---|---|---|---|---|---|---|
| | | ALGA0023779 | 4 | 18464440 | 5.44E-04 | *SNTB1* | NA |
| | | MARC0021142 | 4 | 121957530 | 5.46E-04 | *ENSSSCG00000050922* | NA |
| ADFI | 6.16E-04 | **ALGA0065251** | 12 | 14839808 | 5.60E-05 | ***TEX2***<br>***ICAM2*** | lipid transport process<br>insulin metabolic process |
| | | **ALGA0049005** | 8 | 105105852 | 7.47E-05 | *PRSS12* | zymogen activation |
| | | WU_10.2_4_132878400 | 4 | 121005694 | 1.99E-04 | *PTBP2* | regulation of neural precursor cell proliferation |
| | | **ASGA0084451** | 2 | 31158784 | 2.67E-04 | *ENSSSCG00000034018* | translation |
| | | **ASGA0039519** | 8 | 105054796 | 3.06E-04 | *METTL14* | forebrain radial glial cell differentiation |
| | | ASGA0093786 | 4 | 121249272 | 3.09E-04 | *ENSSSCG00000049773* | NA |
| | | **WU_10.2_6_122065838** | 6 | 131840097 | 3.46E-04 | *ADGRL2* | cell surface receptor signaling pathway |
| | | WU_10.2_4_132842339 | 4 | 120993942 | 3.49E-04 | *PTBP2* | regulation of neural precursor cell proliferation |
| | | **WU_10.2_8_113454144** | 8 | 105564831 | 3.55E-04 | *ENSSSCG00000009116* | Rab protein signal transduction |
| | | **WU_10.2_4_116973174** | 4 | 106826499 | 3.63E-04 | *PHTF1* | signal transduction |
| | | **M1GA0012018** | 8 | 104924079 | 3.87E-04 | *SEC24D* | COPII-coated vesicle cargo loading |
| | | 4_133002201 | 4 | 121131168 | 4.07E-04 | *U6* | NA |
| | | **WU_10.2_9_141498530** | 9 | 128772045 | 4.91E-04 | *KCNK2* | G protein-coupled receptor signaling pathway |
| | | **ASGA0100809** | 9 | 129091764 | 4.96E-04 | ENSSSCG00000015023 | mannosylation |
| | | WU_10.2_4_116864084 | 4 | 106658685 | 5.38E-04 | *AP4B1* | regulation of apoptotic process |
| | | ASGA0022698 | 4 | 120818459 | 6.01E-04 | *ENSSSCG00000043509* | NA |
| | | ALGA0019602 | 3 | 68298863 | 6.14E-04 | ***HK2*** | glucose metabolic process; glycolytic process |
| RFI | 5.89E-04 | WU_10.2_2_139099797 | 2 | 133780841 | 1.77E-04 | *ENSSSCG00000014267* | signal transduction |
| | | **WU_10.2_6_122065838** | 6 | 131840097 | 2.62E-04 | *ADGRL2* | cell surface receptor signaling pathway |
| | | **WU_10.2_1_18721969** | 1 | 16417185 | 2.77E-04 | *PPIL4* | protein peptidyl-prolyl isomerization |
| | | ALGA0049005 | 8 | 105105852 | 2.90E-04 | *PRSS12* | zymogen activation |

| | | | | | |
|---|---|---|---|---|---|
| <u>ALGA0019602</u> | 3 | 68298863 | 3.47E-04 | ***HK2*** | glucose metabolic process; glycolytic process |
| WU_10.2_9_123609853 | 9 | 112285807 | 3.90E-04 | *ENSSSCG00000045606* | NA |
| WU_10.2_9_123578317 | 9 | 112254271 | 4.03E-04 | | |
| WU_10.2_11_4727497 | 11 | 5092611 | 4.76E-04 | ***PDX1*** | glucose metabolic process; insulin secretion |
| MARC0071589 | 9 | 102094613 | 5.27E-04 | *MAGI2* | positive regulation of signal transduction |
| WU_10.2_9_123469671 | 9 | 112313852 | 5.50E-04 | *ENSSSCG00000045606* | NA |
| **ALGA0060467** | 11 | 5379554 | 5.66E-04 | ***PDX1*** | glucose metabolic process; insulin secretion |

[1]FCR = feed conversion ratio; ADG = average daily gain; ADFI = average daily feed intake; RFI = residual feed intake.

[2]$P_{(empirical)}$ _value = the empirical p-values of a multiple testing based on FDR adjusted at the genome-wide significance level of 0.01.

The bold data in SNP name column represents common SNPs identified by linear model GWAS (LM-GWAS) and single-step GWAS (ssGWAS) methods.

The underscore data in SNP name column represents common SNPs related to ADFI and RFI traits.

The bold data in Gene column represents genes related to four feed efficiency related traits.
